# Differences in tissue distribution ability of evodiamine and dehydroevodiamine are due to the dihedral angle of the molecule stereo-structure

**DOI:** 10.3389/fphar.2023.1109279

**Published:** 2023-04-06

**Authors:** Jie Luo, Wen Wen, Jie Chen, Xiaobo Zeng, Ping Wang, Shijun Xu

**Affiliations:** ^1^ School of Pharmacy, Chengdu University of Traditional Chinese Medicine, Chengdu, China; ^2^ Institute of Meterial Medica Integration and Transformation for Brain Disorders, Chengdu University of Traditional Chinese Medicine, Chengdu, Sichuan, China

**Keywords:** alkaloids, transport, tissue distribution, dihedral angle, structure-pharmacokinetics

## Abstract

**Introduction:** This researcher focused at the evodiamine and dehydroevodiamine tissue distribution and structure-pharmacokinetics (PK) relationship after intravenous injection in mice.

**Methods:** Using a transmembrane transport experiment, the permeability of evodiamine and dehydroevodiamine on Caco-2 cells was evaluated. The tissue distribution and pharmacokinetics (PK) of evodiamine and dehydroevodiamine in mice were studied. To comprehend the connection between structure and tissue distribution, physicochemical property evaluations and molecular electrostatic potential (MEP) calculations were performed.

**Results:** Dehydroevodiamine’s Papp values in vitro were 10^−5^ cm/s, whereas evodiamine’s were 10^−6^ cm/s. At a dose of 5 mg/kg, the brain concentration of dehydroevodiamine was 6.44 times more than that of evodiamine. By MEP or physicochemical measures, the permeability difference between evodiamine and dehydroevodiamine is unaffected. The dihedral angle of the stereo-structure appears to be the main cause of the difference in tissue distribution ability between evodiamine and dehydroevodiamine.

**Discussion:** Dehydroevodiamine has a dihedral angle of 3.71° compared to 82.34° for evodiamine. Dehydroevodiamine can more easily pass through the phospholipid bilayer than evodiamine because it has a more planar stereo-structure. Dehydroevodiamine is therefore more likely to pass cross the blood-brain barrier and enter the brain in a tissue-specific manner.

## Graphical Abstract

**Figure:**
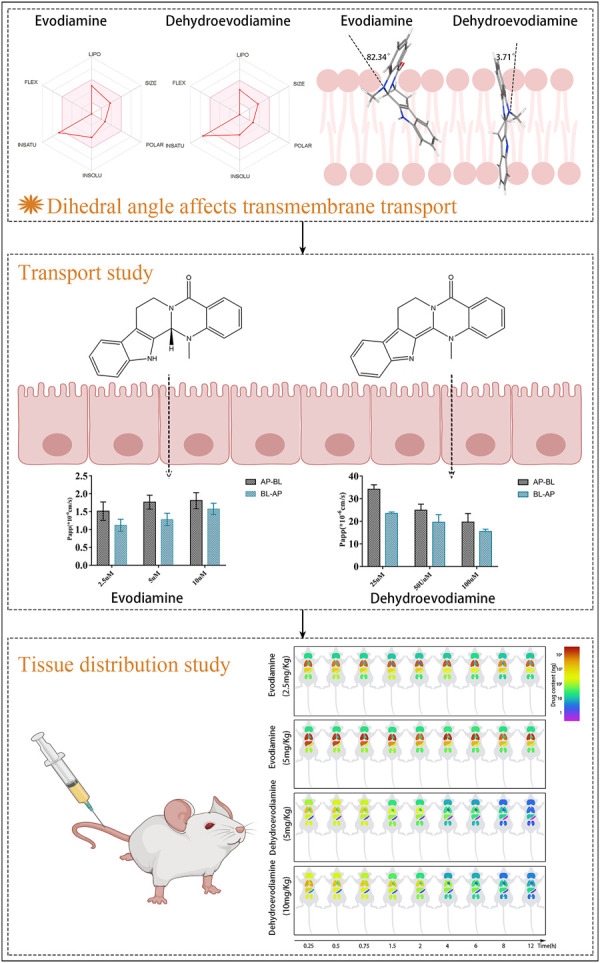
The influence of the dihedral angle of the molecule stereo-structure of evodiamine and dehydroevodiamine on their permeability and tissue distribution.

## 1 Introduction

A drug’s tissue distribution is an important step in getting it to the target tissue or organ from the site of administration and exerting its effect. New medications and formulations with good distribution characteristics may be created by understanding the distribution characteristics of pharmaceuticals *in vivo* and the many contributing circumstances, so that drug can be selectively transported to the desired target organ and stay at the site of action for a long enough time to assure a high degree of efficacy. To guarantee a high level of safety, it is also possible to minimize the distribution to other unneeded organs and reduce the hazardous consequences.

Transmembrane transport is generally understood to be an important component of medication distribution in tissues. The most important aspects of a drug’s physicochemical nature, in addition to the composition of the membrane, determine how a drug may be delivered. Since Lipinski published the “Rule of Five” (RO5) in his landmark paper (Lipinski et al., 2001), the medicinal chemistry community has given greater attention to the physical features of prospective drug candidates. Additional molecular physical features, such as topological polar surface area (PSA) and rotatable bonds, were also identified, and these qualities play a role in the transition of compounds from the pre-exploration to the drug state (Veber et al., 2002). These qualities are not only related to pharmacodynamics, but they are also commonly employed in ADME prediction models. PSA and oral bioavailability (FA) were shown to have a significant sigmoidal association (RMSE = 9.2%, r^2^ = 0.94) (Palm et al., 1997). According to the sigmoidal relationship, medications with a PSA_d_ of less than 63 Å^2^ will be totally absorbed (FA > 90%), whereas those with a PSA_d_ of more than 139 Å^2^ will only be 10% absorbed. The logarithm of the brain/blood concentration ratios, however, showed a substantial connection with PSA (r = 0.917) (Kelder et al., 1999). According to the findings, medicines that are CNS active when taken orally should have a polar surface area of 63.2. The ideal window for medication absorption is widely agreed to be between a log *p*-value of 0 and 3 (Waring, 2009). Poor transmission performance may be caused by values that are either too high (>6) or too low (<−3). Low absorption rates are produced by compounds with high log *p* values because they are poorly soluble in water and spend a lot of time in the lipophilic area. In contrast, very polar substances' poor lipophilicity prevents them from penetrating membrane barriers. Therefore, as part of the compound testing process for the ADME prediction model, characteristics including molecular weight (MW), PSA_d_, rotatable bonds, hydrogen bond donors, and hydrogen bond acceptors are thoroughly scrutinized (Martin, 2005; Pajouhesh and Lenz, 2005; Gleeson, 2008).

**FIGURE 1:**
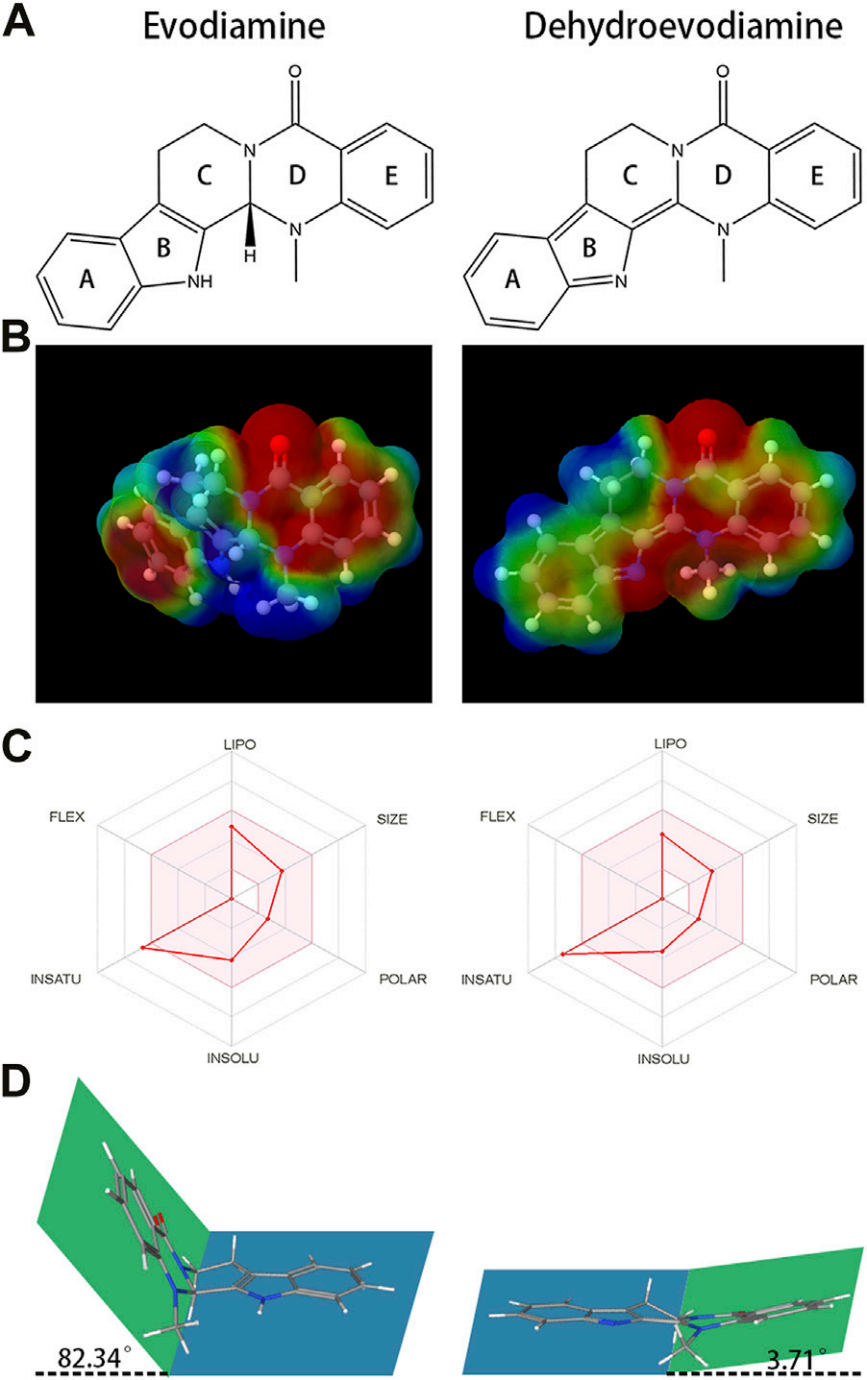
The chemical structures and Molecular electrostatic potential (MEP) of evodiamine (EDM) and dehydroevodiamine (DEDM). **(A)** The chemical structures of evodiamine (EDM) and dehydroevodiamine (DEDM). **(B)** Molecular electrostatic potential (MEP) of EDM and DEDM. Different colors, i.e., Blue, Red, Green, Light Blue, and Light yellow represent the scale of MEP for electron-rich, partial negative charge; electron-deficient, partial positive charge; neutral, uncharged; slightly electron-rich; and slightly electron-deficient respectively. **(C)** Bioavailability Radar of (EDM) and (DEDM). Bioavailability Radar is shown for rapid assessment of drug-likeness. The optimal range for each property is represented by the pink area. Lipophilicity = XLOGP3 +5.0 and −0.7; size = MW between 150–500 g/mol; polarity = TPSA between 20–130 Å^2^; solubility = log S ≯ 6, saturation = fraction of carbons in sp^3^ hybridization ≮ 0.25, and flexibility = rotatable bonds should not be more than nine. **(D)**. The stereostructure of EDM and DEDM (Blue and green represent the two planes of the alkaloid’s stereo structure. Rings A, B and C in the blue plane, rings D and E in the green plane. The dihedral angle is 82.34° for EDM and 3.71° for DEDM.

Indole alkaloids, one of the most important natural alkaloids, synthesised from tryptophan or tryptamine as precursors, possess a bicyclic skeleton consisting of a benzene ring fused to a five-membered pyrrole ring (Rosales et al., 2020). Up to now, more than 4,000 different indole alkaloids have been identified. Evodiamine (EDM) and dehydroevodiamine (DEDM) (Figure 1A) are two representative indoloquinazoline alkaloids extracted from *Evodia Rutaecarpa*, and have been reported to be of anti-inflammatory (Choi et al., 2006; Hu et al., 2015), anti-cancer (Nejak-Bowen and Monga, 2011; Wang et al., 2020; Lin and Yeh, 2021), hepatorenal protective (Yang et al., 2018), cardioprotective (Zhu et al., 2011; Tian et al., 2019) et al. EDM and DEDM have the same core structure, except for slightly different substituents. The outcomes reveal that the oral bioavailability of EDM is 0.1%, and that of DEDM is 15.35% (Lin et al., 2012). These differences may be related to many factors, such as polarity, lipophilicity and molecular stereostructure. Elucidating the link between drug physical qualities, stereostructure, and tissue distribution can assist to identify a strategy to adjust the structure to maximize pharmacological attributes, laying the groundwork for the creation of novel medications.

The permeability of EDM and DEDM in the Caco-2 monolayer cell membrane was investigated first, followed by tissue distribution of these two compounds in mice following intravenous injection. Following the collection of permeability and tissue distribution ability parameters, it was determined which structural parameter was the key factor influencing ADME performance by comparing their differences of the physical characteristics, molecular electrostatic potentiasl (MPF), and stereo-structures.

## 2 Materials and methods

### 2.1 Materials

EDM (EDM >99%) and DEDM (DEDM >99%) were purchased from Chengdu Alfa Biotechnology Co., Ltd. (Chengdu, China). Acetonitrile (HPLC grade) was got from fisher chemical (Massachusetts, United States). Non-essential amino acids solution from Sigma-Aldrich (Shanghai, China) and Hank’s balanced salt solution (HBSS), Penicillin-Streptomycin Solution (×100) and MEM were purchased from Hyclone (Massachusetts, United States). Sodium pyruvate was bought from solarbio science and technology Co., Ltd., (Beijing, China). Gibco (New York State, United States) supplied the 0.25% Trypsin-EDTA and penicillin-streptomycin solution. From Every Green Biotechnology Co., Ltd., (Zhejiang, China), FBS was bought. DMSO was obtained from Meilunbio (Dalian, China). 12-well Transwell^®^ plate was acquired from Corning (New York State, United States). The preparation of aqueous solutions uses double-distilled water and all additional solvents are analytical agents.

### 2.2 Cell culture

The Center of Cellular Resources at the Chinese Academy of Sciences (Shanghai, China) provided the human colon adenocarcinoma cell line Caco-2, and the experiment utilized 15–30 generations of these cells. DMEM enriched with 15% FBS, 1% non-essential amino acids, 1% Penicillin-Streptomycin Solution (×100), and 1% sodium pyruvate was utilized to cultivate cells at 37°C in a humidified atmosphere of 5% CO_2_. The cells were separated from flasks when they achieved the anticipated confluence threshold (70%–80%), and they were then inoculated in 12-well plates at a density of 2–5 × 10^5^ cells/cm^2^ in a polycarbonate Transwell^®^ (pore size: 0.4 µm; filter area: 1.12 cm^2^). In the first week, the culture media was switched every 2 days. After that, the culture medium was switched out daily until the transepithelial electrical resistance (TEER) for transport experiments reached 200 Ω/cm^2^; It has been demonstrated in our prior experiments that this process requires 18–21 days under the specified culture conditions (Huang et al., 2020).

### 2.3 Cell viability assay

The cytotoxicity of EDM and DEDM was assessed in Caco-2 cells using the aforementioned MTT test. These alkaloids were produced as solutions in DMSO and then diluted in cell culture media. Inoculating Caco-2 cells (1 × 10^4^cells/well) in 96-well plates with 200 μL of media. After additional 48 h of incubation with EDM and DEDM (final test alkaloids concentrations ranged from 0.4 to 200 μM, and DMSO was less than 0.05%), the medium was discarded. To each well, an aliquot of 190 μL freshly prepared DMEM (serum-free) having 10.0 μL MTT (5 mg/mL) was added. The medium in every well was then removed from the plate after a further 4 h at 37°C of incubation. In 100 μL of DMSO, the purple formazan product was dissolved. Using a microplate reader, the ratio of absorbance (treated to untreated cells) at 490 nm was employed to measure cell viability.

### 2.4 Cell transport assay

Caco-2 monolayers were pre-cultured in HBSS for 15 min at 37°C after being rinsed two times with preheated Hanks' balanced salt solution (HBSS, pH = 7.4). The donor side was supplemented with DEDM (25, 50, and 100 μM) and EDM (2.5, 5, and 10 μM), and the receiver chamber was filled with the corresponding volume of HBSS medium. For 120 min, the plate was incubated at 37°C. At 0, 30, 45, 60, 90, and 120 min, a 0.5 mL aliquot of each receiver chamber’s sample was taken, and 0.5 mL of HBSS was added to keep the volume constant. By using high-performance liquid chromatography (HPLC), the samples were examined. The sample (300 μL) taken from the receiver chamber was mixed with 300 μL of acetonitrile, and the combination was centrifuged for 10 min at 13,000 rpm. Lastly, a 100 µL aliquot of the supernatant was injected into the HPLC for examination.

Two drugs were quantitatively determined using an Agilent 1,260 HPLC apparatus (United States). A C18 column (1.8 μm, 3.0 × 150 mm; Agilent Technologies, Santa Clara, CA, United States) was used for the separation, with a column temperature of 25°C. The mobile phase comprised both A (water) and B (acetonitrile). The ratio between A and B was 70: 30 with a flow rate of 1 mL per minute. For EDM or DEDM, 20 μL samples were injected and analyzed at 290 nm or 344 nm, respectively. Acetonitrile was used to create the standard stock solutions for EDM and DEDM, respectively. By serially diluting standard stock solutions with HBSS, working solutions were made. The effective concentrations were 0.04–2.5 μg/mL for EDM and 0.23–7.5 μg/mL for DEDM, respectively. These samples were stored at 4°C before being used.

Eqs 1, 2 were used to determine the apparent permeability coefficients (Papp) and efflux ratio (ER) of marker drugs:

$$P_{app}=dQ/\left({dtC}_{0}A\right)$$ (1)

where dQ/dt is the cumulative transport rate (M/min), C_0_ is the initial concentration of each marker drug, and A is the surface area of the filters.

$$ER=P_{app\left(BL-AP\right)}/P_{app\left(AP-BL\right)}$$ (2)

Data for transport experiments are presented as the mean ± SD of six independent experiments.

### 2.5 Animal

252 male KM mice (25 ± 2 g) were purchased from the Hunan SJA Laboratory Animal Co., Ltd., China. (China. Certificate: SYXK [Xiang] 2019-049). Each mouse was kept in a climate-controlled environment (22°C–28°C) with unrestricted access to food and water and a regular 12 h light/dark cycle. Before the experiment, they spent 2 days adjusting to their housing. All mice used by the Institute of Material Medical Integration and Transformation for Brain Disorders (IBD2020007) received human care in accordance with the standards established by the National Institutes of Health. The Chengdu University of Traditional Chinese Medicine’s research committee approved this project.

### 2.6 Tissue distribution of EDM and DEDM

In a sterile mortar, 2-hydroxypropyl-β-cyclodextrin and EDM or DEDM were added. The mixture was then transferred to a sterile centrifuge tube after being repeatedly ground for 30 min with a tiny amount of ordinary saline added. After that, saline was added and ultrasonically processed for 1 h. In a vacuum freeze drier, the suspension was freeze-dried for 30 h. To make the injection, the freeze-dried powder was ultimately redissolved in regular saline. After acclimatization, all mice were acclimated before being randomly placed into four groups of three each: EDM (H) group, EDM (L) group, DEDM (H) group, DEDM (L) group. Food was discontinued 12 h before the trial. Mice in EDM (H) group and EDM (L) group were injected EDM at the dose of 5 mg/kg or 2.5 mg/kg, and mice in DEDM (H) group and DEDM (L) group were injected DEDM at the dose of 10 mg/kg or 5 mg/kg. The blood was drawn and then promptly placed into a heparinized tube at the time points of 0.25, 0.5, 1, 2, 3, 4, 6, 8 and 12 h after dose (at each time point). Supernatant plasma from the collected sample was transferred into another tube and kept at −80°C until analysis after being centrifuged at 3,000 rpm for 10 min. Immediately after blood was obtained, the mice were dissected. Saline was injected into the heart until the liver turned white. Then, the organs of the heart, liver, spleen, lung, kidney, and brain were removed, weighed, and homogenized (2 mL normal saline per 1 g tissue sample, 30 Hz, 1.5 min) before being kept at - 80°C for analysis. The calibration standards, tissue samples, and plasma samples were pretreated with acetonitrile for protein precipitation. To a 100 µL portion of each of the plasma or tissue sample, 200 µL of acetonitrile with internal standard was added. To remove the denatured protein, the mixture was vortexed for 1 min and then centrifuged at 16,500 rpm for 10 min. The supernatant was finally injected into the LC-MS/MS in an aliquot of 100 µL for analysis.

The Agilent 1,260 HPLC-MS/MS system has a thermostatically controlled column compartment, an online degasser, a quadruple pump, and an auto-sampler. Agilent Eclipse Plus C8 RRHD (3.0 × 150 mm, 1.8 μm) was used to perform the liquid chromatographic separation. Using an isocratic elution, the EDM and DEDM were analyzed. Both A (0.1% formic acid) and B (acetonitrile) were contained in the mobile phase. With a flow rate of 0.3 mL/min, the ratio between A and B was 20: 80 for 4 min. The column temperature was kept at 35°C. Prior to injection, samples were transferred in an auto-sampler at a temperature of 4°C and 5 µL was the injection volume. The EDM and DEDM were quantitated by using an Agilent 1260-6460 mass spectrometer. The ion source was electrospray ionization (ESI) and adopts positive ion mode. The scan mode was multiple reaction monitoring (MRM). The operating conditions for the EDM and DEDM were 4000 V capillary voltage. The dry gas flow was 11 min per liter, and the dry gas temperature was 300°C. Ion source gases 1 (GS 1) and 2 (GS 2) were both set to 15. The EDM and DEDM were detected through MRM modes with m/z transition at 304.3→171 for EDM and 301.3→286 for DEDM (for details, see Supplementary Table S1).

Acetonitrile was used to prepare standard stock solutions for each type of EDM and DEDM individually. By diluting the standard stock solutions serially with acetonitrile, working standards for the EDM and DEDM were made. The calibration curve samples were acquired by spiking the series working standard solutions (10 μL) into a tube and evaporating the solvent, and then adding blank mouse tissue homogenate or plasma (100 μL). Thus, the effective concentrations of EDM and DEDM in tissue homogenate or plasma samples were 1.8–364 ng/mL. These samples were stored at 4°C before being used. DEDM is used as an internal standard compound when detecting EDM. Meanwhile, when DEDM is detected, EDM is used as the internal standard compound. The final concentration of the internal standard compound is 10 ng/mL. The research shows EDM and DEDM do not interconvert *in vivo* (Zhang Z. et al., 2018; Wang et al., 2018), so they can be used as internal standard compounds for each other.

For the Non-compartmental PK analysis, Thermo Kinetica (Version 5.0, Thermo Electron Corporation, Waltham, MA) was employed. Based on observed data, the time when the maximum tissue concentration was reached (T_max_) and the maximum tissue concentration (C_max_) were assessed. By using the linear trapezoidal rule, the area under the plasma-concentration-time curve was calculated from 0 h to infinity (AUC_0-∞_) and from 0 h to 12 h. Additionally, the mean residence time (MRT) was computed. PK parameters are reported as the mean of seven independent experiments. Linear and non-linear regressions were analyzed in Microsoft Excel 2016^®^ (United States).

### 2.7 Physicochemical information of alkaloids

Drug potency is largely determined by its physicochemical qualities. SwissADME is a free online tool for assessing small molecule pharmacokinetics, drug-likeness, and medicinal chemistry friendliness (Daina et al., 2014; Daina and Zoete, 2016; Daina et al., 2017). We obtained EDM and DEDM physical representations from the SwissADME version 15 database.

### 2.8 Molecular electrostatic potential calculation

Electrostatic interactions, as a key feature of intermolecular non-covalent interactions, significantly affect the permeability of compounds. Intermolecular electrostatic interactions are often described accurately by molecular electrostatic potential (MEP) surfaces. PubChem was used to get the two indole alkaloids' structures (Kim et al., 2016). Using the AM1-BBC technique, the molecules' partial charges were added in Chimera (Pettersen et al., 2004). The MEP values were calculated using the adaptive Poisson-Boltzmann solver (APBS) (Baker et al., 2001). The dielectric coefficients of the compound and solvent were set to 2 and 78.5, respectively and all other parameters were set to default values. Molecular surfaces were visualized employing Chimera (Chimera version 1.12).

## 3 Results

### 3.1 Transmembrane transport of alkaloids

Cell morphology was examined under a microscope over the course of the 21 days. Results indicated that the cell boundary is clearly defined and that the cell shape is homogeneous, compact, and flat. The experimental conditions were met by the final resistance TEER, which is larger than 500/cm^2^, showing that the model is appropriate for transport study. The HBSS chromatogram can be compared to the matching spiked samples' chromatogram to demonstrate the method’s high selectivity. No obvious HBSS interference was seen for the two identified drugs, as shown in Supplementary Figure S1. For EDM and DEDM, the retention times were 4.067 and 5.869 min, respectively. Over the concentration range of 0.04–2.5 g/mL for EDM and 0.23–7.5 g/mL for DEDM, the calibration curves were linear (see Supplementary Table S2 for details). The lowest limit of quantification (LLOQ) for EDM was 0.03 g/mL and 0.2 g/mL for DEDM, and the signal-to-noise ratios were greater than 10. The average recoveries for the three concentrations range between 90% and 110%. Both EDM and DEDM’s intra-day and inter-day accuracy (RSD,%) were under 15.0.

**FIGURE 2:**
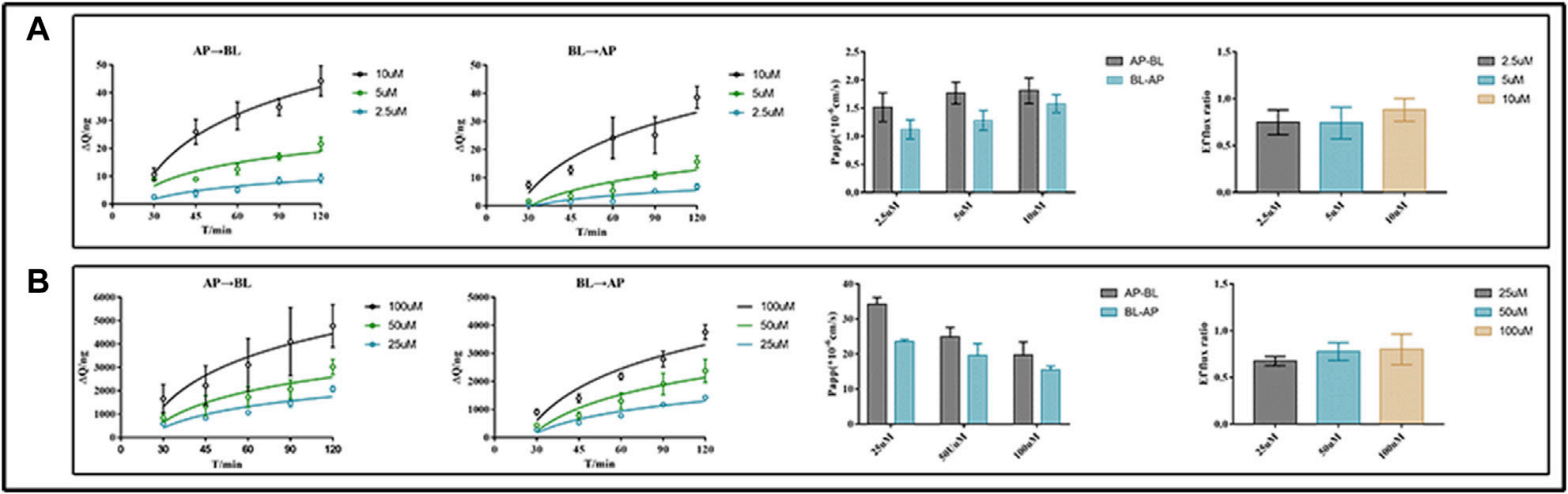
Transmembrane transport studies of evodiamine (EDM) and dehydroevodiamine (DEDM). CaCo-2 cells were cultured in transwell plate for 21 days to form Caco-2 monolayers. The EDM (2.5, 5, and 10 μM) and DEDM (25, 50, and 100 μM) were added to the donor side and the HBSS medium was placed in the receiver chamber. The sample was collected at 0, 30, 45, 60, 90, and 120 min from each receiver chamber. High-performance liquid chromatography (HPLC) was used to analyze samples and assess the efflux ratio (ER) and calculate apparent permeability coefficients (P_*app*_). The accumulation and P_*app*_ of drugs across AP→BL and BL→AP, as well as the efflux ratio of drugs, are shown from left to right. **(A)** EDM; **(B)** DEDM. Data are shown as the mean ± SD (*n* = 6).

For the transport studies, we used EDM (2.5, 5, and 10 μM) and DEDM (25, 50, and 100 μM) based on the findings of the non-toxic concentration trials (for more information, see Supplementary Table S3). Bidirectional tests, as shown in Figure 2, revealed transmembrane drug transport across Caco-2 cell monolayers. The P_*app*_ value of DEDM found in the current investigation was at the level of 10^−5^ cm/s and is attributed to compounds with high permeability. However, EDM’s P_*app*_ values were at a level that is regarded as having medium permeability (10^–6^ cm/s). Comparing bidirectional transport for EDM and HEDM reveals that the permeability in the secretory direction (basolateral→apical) and the absorptive direction (apical→basolateral) were nearly equal.

### 3.2 Tissue distribution of EDM and DEDM

**FIGURE 3:**
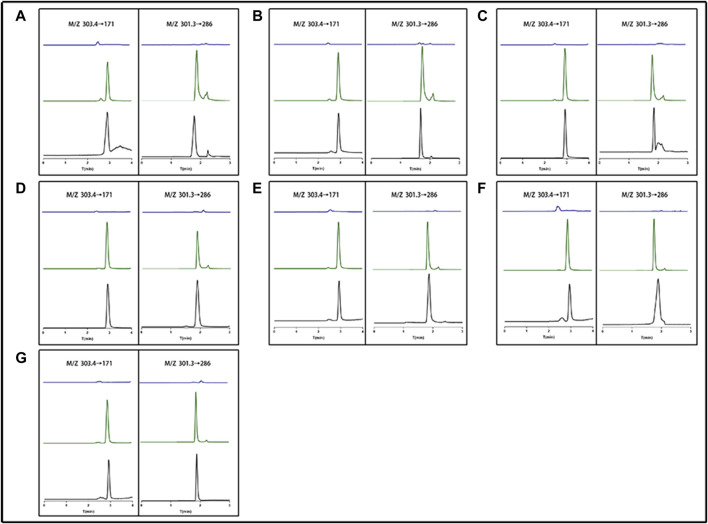
The representative chromatogram of evodiamine (EDM) and dehydroevodiamine (DEDM). **(A)** Heart; **(B)** Liver; **(C)** Spleen; **(D)** Lung; **(E)** Kindy; **(F)** Brain; **(G)** Plasma; The left side (M/Z 303.4→171) represents EDM; The right side (M/Z 301.3→286) represents DEDM; blue for blank tissue homogenate or blank plasma; Green for standard plus tissue homogenate or plasma; black for tissue homogenate or plasma sample.

By contrasting the chromatograms of blank tissue homogenates or plasma samples taken from six drug-free mice with the matching spiked tissue homogenates or plasma samples, the method’s selectivity was evaluated. Figure 3 demonstrates that neither mouse tissue homogenates nor plasma significantly influenced the retention periods of either analyte. EDM and DEDM had retention times of 2.919 and 1.814 min, respectively. EDM and DEDM calibration curves were linear for the 1.8–364 ng/mL concentration range (see Supplementary Tables S4, S5 for details). The lower limit of quantification (LLOQ) was 0.9 ng/mL with a signal/noise ratio greater than 10. The signal to noise ratio (SNR) of EDMs and DEDMs was greater than 10, and the lower limit of quantification (LLOQ) was 0.9 ng/mL. For the three concentrations, the average recovery of mouse tissue homogenates or plasma ranged from 90% to 110%. For both EDM and DEDM, the intra-day and inter-day precision (RSD,%) was under 15.0. The mean IS-normalized matrix factor of the three concentrations was over than 93.7% in plasma and tissues. The results demonstrated that this quantitative approach is capable of detecting two alkaloids quantitatively in plasma and tissues. The analyses were carried out at room temperature (20°C–24°C) in triplicate.

**FIGURE 4:**
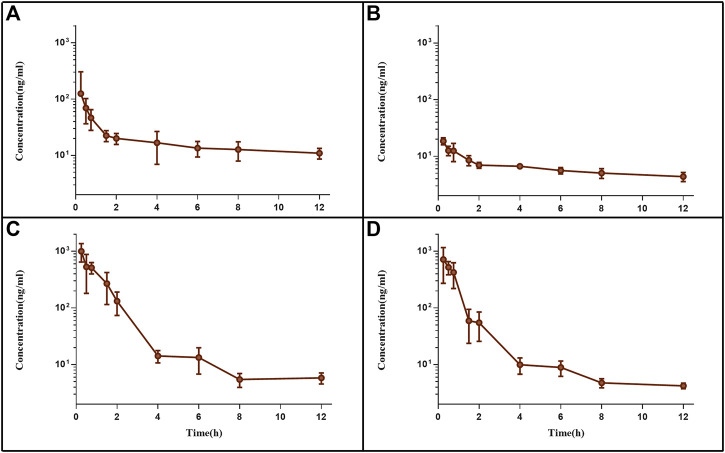
The plasma pharmacokinetic curves of evodiamine (EDM) and dehydroevodiamine (DEDM). The injection of EDM (5, 2.5 mg/kg) and DEDM (10, 5 mg/kg) was injected through tail vein of mice. Blood was collected at 0.25, 0.5, 1, 2, 3, 4, 6, 8, and 12 h after the dose (with 7 mice at each time point). The collected samples were centrifuged for 10 min at 3,000 rpm and plasma from supernatants was transferred to other tubes. The plasma samples were analysed by HPLC-MS/MS and draw pharmacokinetic curves. **(A)** EDM (5 mg/kg); **(B)** EDM (2.5 mg/kg); **(C)** DEDM (10 mg/kg); **(D)** DEDM (5 mg/kg); Data are shown as the mean + SD, *n* = 7.

**FIGURE 5:**
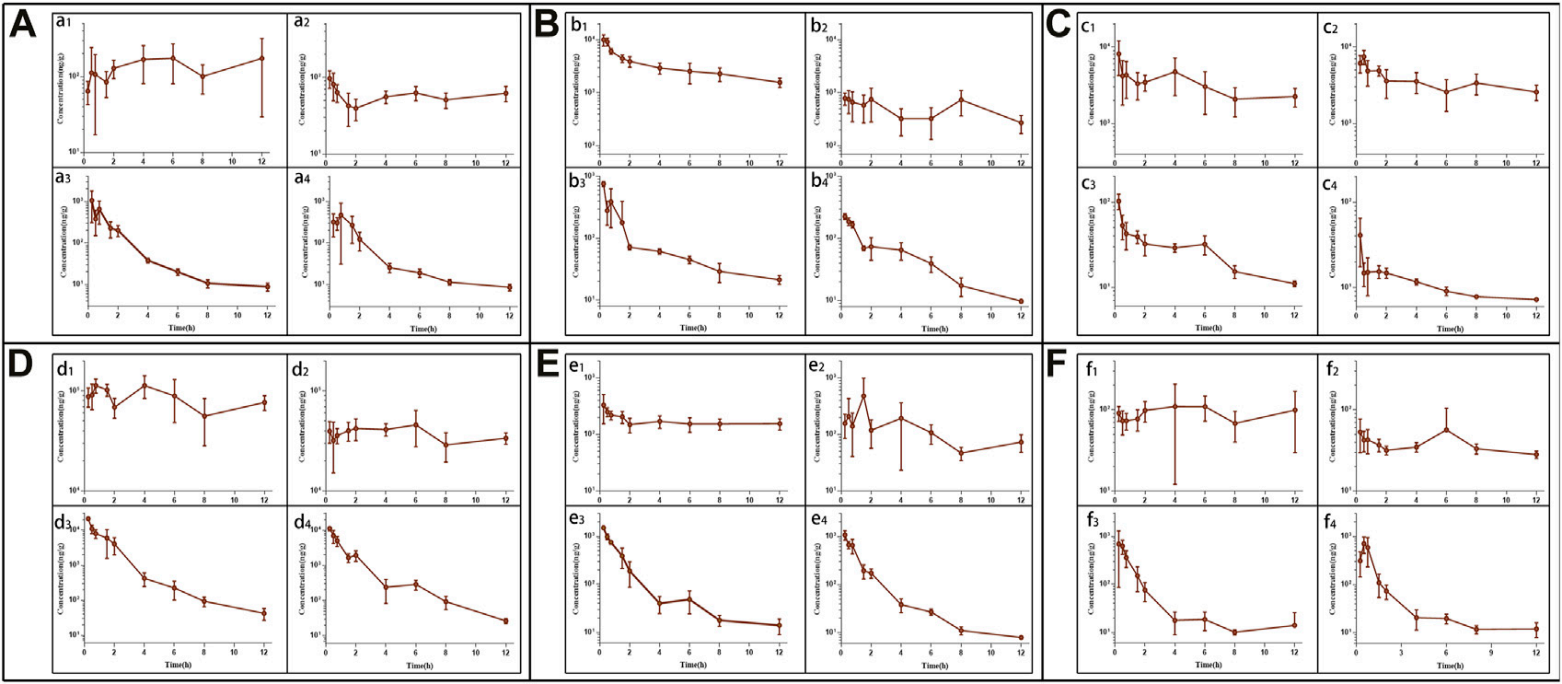
The tissue pharmacokinetic curves of evodiamine (EDM) and dehydroevodiamine (DEDM). The EDM (5, 2.5 mg/kg) and DEDM (10, 5 mg/kg) was injected through tail vein. After blood was collected at 0.25, 0.5, 1, 2, 3, 4, 6, 8 and 12 h after dose (with 7 mice at each time point), the mice were rapidly dissected. The heart was perfused with saline until the liver became white. Next, the heart, liver, spleen, lung, kidney, and brain were excised, weighed and homogenised. The tissue samples were analysed by HPLC-MS/MS and draw pharmacokinetic curves. **(A)** Heart; **(B)** Liver; **(C)** Spleen; **(D)** Lung; **(E)** Kindy; **(F)** Brain; a_1_ EDM (5 mg/kg); a_2_ EDM (2.5 mg/kg); a_3_ DEDM (10 mg/kg); a_4_ DEDM (5 mg/kg); The rest of the tissues are the same as the heart. Data are shown as the mean + SD, *n* = 7.

**FIGURE 6:**
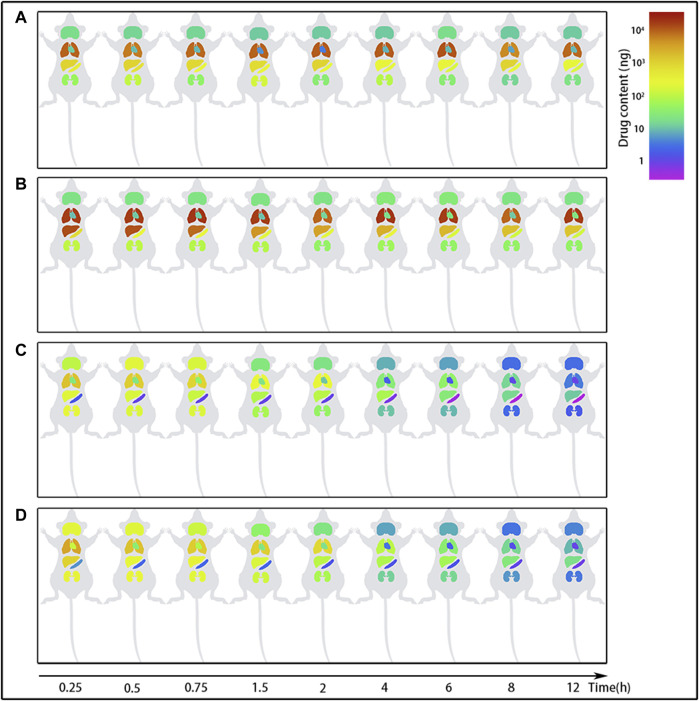
The content changes of evodiamine (EDM) and dehydroevodiamine (DEDM) in the organs of mice. EDM (5, 2.5 mg/kg) and DEDM (10, 5 mg/kg) were administered to mice *via* the tail vein. The content of alkaloids in tissue was calculated by multiplying tissue concentration (at each time point) by the corresponding tissue weight. Tissue was collected at time points of 0.25, 0.5, 1, 2, 3, 4, 6, 8 and 12 h after dose (at each time point with 7 mice). Tissue concentration of alkaloids was then analyzed by HPLC-MS/MS. The mean (*n* = 7) is provided for the following data: **(A)** EDM (2.5 mg/kg); **(B)** EDM (5 mg/kg); **(C)** DEDM (5 mg/kg); and **(D)** DEDM (10 mg/kg).

Following tail vein injection, the concentrations of EDM and DEDM in mice’s plasma (Figure 4) and tissue (Figure 5) as well as the primary pharmacokinetic characteristics of EDM and DEDM (Table 1) were displayed. Figure 6 depicts the variations in EDM and DEDM content in several mouse organs. These findings demonstrated that the plasma concentration of EDM and DEDM reached its maximum concentration (C_max_) within 15 min and then began to exponentially decline. After reaching its peak concentration in 15 min, the distribution of DEDM in different tissues falls exponentially, and the half-life (T_1/2_) and mean residence time (MRT) of EDM in different tissues are longer. Both EDM and DEDM were found in the brain, with DEDM having a 6.44 times higher drug concentration than EDM at a dose of 5 mg/kg. Both EDM and DEDM were observed to cause pulmonary enrichment, reaching maximum concentrations of 113.757 μg/g for EDM and 11.267 μg/g for DEDM at doses of 5 mg/kg. Second, the order of EDM tissue distribution concentration is kidney, brain, heart, liver, and spleen, whereas DEDM tissue distribution concentration is kidney, heart, brain, liver, and spleen. The distribution of EDM in the liver, spleen, and lung was significantly higher than that of DEDM at the same dose (5 mg/kg). In contrast to DEDM, EDM was less widely distributed in the heart, kidney, and brain.

**TABLE 1:** PK parameters of evodiamine and dehydroevodiamine.

Tissue	Drug	Dose (mg/kg)		Parameter					
				C_max_ (μg/g)	T_max_ (h)	AUC_0-t_ (μg/g*h)	AUC_0-∞_ (μg/g*h)	T_1/2_ (h)	MRT (h)
Heart	evodiamine	2.5	min	0.074	0.25	0.48	0.48	—	6.25
			max	0.137	0.25	0.96	0.96	—	5.92
			average	0.097	0.25	0.68	0.68	—	6.02
		5	min	0.088	2.00	0.61	0.61	—	6.16
			max	0.478	12.00	3.34	3.34	—	6.73
			average	0.176	6.00	1.66	1.66	—	6.36
	dehydroevodiamine	5	min	0.198	0.50	0.39	0.46	7.20	5.71
			max	1.394	0.75	1.81	1.88	5.00	2.28
			average	0.472	0.75	0.87	0.93	4.97	3.04
		10	min	0.483	0.25	0.60	0.61	2.13	2.37
			max	2.498	0.25	2.34	2.35	1.62	1.35
			average	1.051	0.25	1.24	1.25	1.66	1.76
Liver	evodiamine	2.5	min	0.446	0.25	2.31	4.51	12.06	17.22
			max	1.611	2.00	11.01	17.91	9.10	13.06
			average	0.792	0.25	5.99	10.19	9.66	13.97
		5	min	6.670	0.50	21.68	24.63	3.89	5.66
			max	13.238	0.25	48.86	77.95	9.29	12.32
			average	10.128	0.25	37.94	58.48	8.84	11.57
	dehydroevodiamine	5	min	0.200	0.25	0.45	0.50	3.84	4.38
			max	0.274	0.25	0.88	0.91	2.42	3.88
			average	0.226	0.25	0.62	0.67	3.13	4.05
		10	min	0.690	0.25	0.71	0.79	4.28	4.35
			max	0.888	0.25	1.73	1.95	5.89	4.33
			average	0.758	0.25	1.07	1.23	5.36	4.84
Spleen	evodiamine	2.5	min	5.406	0.50	25.83	84.05	23.78	33.72
			max	10.374	0.50	62.85	126.60	12.55	17.75
			average	7.473	0.50	41.71	71.73	9.30	13.73
		5	min	3.575	0.25	16.63	16.63	—	5.32
			max	15.351	0.25	65.31	88.98	5.80	9.01
			average	8.140	0.25	38.41	74.00	11.73	16.53
	dehydroevodiamine	5	min	0.018	0.25	0.11	0.62	50.33	70.61
			max	0.083	0.25	0.16	0.23	7.55	9.94
			average	0.041	0.25	0.13	0.22	9.19	12.91
		10	min	0.070	0.25	0.25	0.34	6.69	9.16
			max	0.128	0.25	0.42	0.51	4.84	6.77
			average	0.102	0.25	0.33	0.42	5.59	7.61
Lung	evodiamine	2.5	min	36.478	4.00	272.17	272.17	—	5.78
			max	72.665	6.00	621.61	1100.85	8.58	13.84
			average	45.866	6.00	445.42	1676.98	26.42	38.32
		5	min	82.557	0.75	518.94	518.94	—	5.54
			max	140.170	4.00	1370.46	3,012.72	12.55	19.05
			average	113.757	0.75	986.45	3,159.59	22.05	31.88
	dehydroevodiamine	5	min	9.137	0.25	7.72	7.77	2.06	1.37
			max	14.031	0.25	19.30	19.37	1.68	1.45
			average	11.267	0.25	12.98	13.04	1.79	1.42
		10	min	18.697	0.25	13.57	13.58	1.17	0.92
			max	23.885	0.25	37.33	37.38	1.29	1.40
			average	21.241	0.25	23.65	23.78	2.39	1.33
Kindy	evodiamine	2.5	min	0.067	0.50	0.51	1.30	16.13	23.75
			max	1.364	1.50	3.22	3.58	3.70	5.20
			average	0.481	1.50	1.48	1.80	4.63	6.98
		5	min	0.173	0.75	1.36	1.36	—	5.80
			max	0.591	0.25	2.87	9.66	24.06	34.45
			average	0.332	0.25	2.04	2.04	—	5.56
	dehydroevodiamine	5	min	0.909	0.25	0.99	1.00	1.89	1.72
			max	1.590	0.25	1.88	1.90	2.02	1.66
			average	1.075	0.25	1.34	1.36	2.18	1.73
		10	min	1.359	0.25	1.37	1.42	4.08	1.81
			max	1.701	0.25	2.50	2.53	1.84	2.04
			average	1.508	0.25	1.87	1.89	1.71	1.71
Brain	evodiamine	2.5	min	0.029	0.50	0.32	1.61	34.89	50.99
			max	0.165	6.00	0.72	0.84	3.01	7.26
			average	0.057	6.00	0.45	0.70	6.53	11.30
		5	min	0.068	2.00	0.60	0.60	—	5.70
			max	0.332	4.00	2.08	2.08	—	6.22
			average	0.110	4.00	1.10	1.10	—	6.00
	dehydroevodiamine	5	min	0.473	0.50	0.43	0.47	4.32	3.69
			max	1.230	0.50	1.56	1.58	1.90	1.83
			average	0.708	0.50	0.87	0.88	1.99	2.02
		10	min	0.306	0.50	0.39	0.53	12.40	9.33
			max	2.022	0.25	1.79	1.81	2.01	1.87
			average	0.694	0.25	0.93	0.94	1.94	1.83
Blood	evodiamine	2.5	min	0.017	0.25	0.06	0.09	8.03	10.90
			max	0.024	0.25	0.10	0.27	22.42	29.96
			average	0.019	0.25	0.08	0.19	17.30	23.50
		5	min	0.045	0.25	0.12	0.12	—	4.38
			max	0.532	0.25	0.55	0.74	9.64	8.53
			average	0.125	0.25	0.25	0.56	19.51	23.77
	dehydroevodiamine	5	min	0.300	0.50	0.33	0.33	1.86	1.78
			max	1.448	0.25	1.29	1.29	1.43	1.12
			average	0.717	0.25	0.74	0.78	5.99	1.98
		10	min	0.566	0.25	0.45	0.46	2.39	1.83
			max	1.566	0.25	1.93	1.94	1.42	1.24
			average	1.002	0.25	1.14	1.14	1.48	1.28

C_max_, peak tissue or plasma concentration; T_max_, time to reach peak tissue or plasma concentration; AUC_0–t_, area under the tissue or plasma concentration–time curve from 0 to 12 h; AUC_0–∞_, area under the tissue or plasma concentration–time curve from 0 to infinity; T_1/2_, terminal elimination half-life; MRT, mean residence time.

The distribution of EDM in the spleen and kidney showed no dosage difference, indicating that the organs' propensity for saturation with EDM. The largest concentrations of EDM after intravenous injection of 2.5 mg/kg or 5 mg/kg were found in the spleen, where they were 7.473 μg/g and 8.14 μg/g, respectively. The highest concentrations in the kidney were 0.481 μg/g and 0.332 μg/g, respectively. The distribution of DEDM in the kidney (1.075 μg/g or 1.508 μg/g) and brain (0.708 μg/g or 0.694 μg/g) likewise approached saturation after injections of 5 mg/kg or 10 mg/kg DEDM into the tail vein.

### 3.3 Physicochemical information and molecular electrostatic potential surfaces of alkaloids

Table 2 presents physicochemical details of the two indole alkaloids. The SwissAEDM database was used to generate the molecular weights, Log*P* values, hydrogen bond donors and acceptors, topological polar surface areas (tPSAs), and rotatable bonds reported in this table. There was essentially no difference between the alkaloids in terms of molecular weights, Log*P* values, hydrogen bond donors and acceptors, topological polar surface areas (tPSAs), and rotatable bonds. The calculation of atomic charge to determine molecular electrostatic potential (MEP) is an easy way to describe molecule polarity. Figure 1B displays the molecular electrostatic potential (MEP) surfaces of EDM and DEDM, and Supplementary Table S6 displays the atomic charges. The outcomes demonstrated that the two alkaloids have identical atomic charges.

**TABLE 2:** The physicochemical properties of evodiamine and dehydroevodiamine.

Name	Mw (g/mol)	H-bond donor	H-bond acceptor	Log*P*	Rotatable bonds	Topological polar surface area (Å^2^)
Evodiamine	303.36	1	1	2.7	0	39.34
Dehydroevodiamine	301.34	0	2	2.29	0	39.29

The above data are from SwissADME: http://www.swissadme.ch.

The bioavailability radar map displays the computed physicochemical data of compounds in the context of the oral drug-like property space. Figure 1C displays the bioavailability radar for EDM and DEDM. With the exception of saturation, EDM and DEDM’s lipophilicity, size, polarity, and flexibility were all within the ideal range and did not differ noticeably. By utilizing the carbon percentage in sp^3^ hybridization, saturation is a technique for describing the three-dimensional structure of a molecule. As a result, we looked at the stereostructure of two alkaloids and discovered that rings D and E and rings A, B, and C are on opposite planes, creating a dihedral angle (Figures 1A, D).

## 4 Discussion

The results of Caco-2 cell transport demonstrated that DEDM had better membrane permeability than EDM in this study. In a tissue distribution experiment with mice, DEDM was demonstrated to be more easily able to cross the blood-brain barrier than EDM. Due to its smaller dihedral angle than EDM, DEDM has a more planar stereo-structure, is more likely to cross phospholipid bilayers, and has higher permeability. DEDM is therefore more likely to cross the blood-brain barrier (BBB) and reach the brain in a tissue-specific manner.

Six isoquinoline alkaloids with just minor structural differences (see Supplementary Figure S2 for chemical structures) were examined for permeability on Caco-2 cells. The P_*app*_ values of evolitrine (EVT), DEDM, and hydroxyevodiamine (HEDM) observed in the current investigation were at the level of 10^−5^ cm/s, as shown in Supplementary Figure S3. But EDM, rutaecarpine (RUP), and 1-hydroxyrutaecarpine (1-HRUP) all had P_*app*_ values of 10^−6^ cm/s or higher. Bidirectional transport comparisons for EDM, EVT, DEDM, HEDM, and 1-HRUP revealed nearly comparable permeabilities in the secretory (basolateral→apical) and absorptive (apical→basolateral) directions. RUP’s permeability was, however, more than twice as high in the secretory direction as it was in the absorptive direction.

The core molecular structure of these six alkaloids is the same, but due to varying substituents, their molecular polarity, lipid solubility, and other physical and chemical characteristics vary, which affects their penetration, absorption, distribution, and action. The structures of EDM and DEDM are quite similar; DEDM is produced by forming a double bond by removing two hydrogen atoms from the N-14 atom of EDM. However, our research reveals that the permeability of these two molecules differs significantly. Previous research has demonstrated that the N-14 atom’s substituent is a critical factor in the AHR activation of EDM and DEDM and plays a significant role in the link between the structure and activity of indolequinazoline alkaloids (Zhang Y. et al., 2018). Therefore, EDM and DEDM were chosen to investigate the distribution of their tissues and differences *in vivo*.

In tissue distribution tests, lung targeting was seen with both EDM and DEDM. The AUC_0-t_ of EDM at the same dose (5 mg/kg) was 986.45 μg/g*h in the lung, 1.16 μg/g*h in the heart, 37.94 μg/g*h in the liver, 38.41 μg/g*h in the spleen, 2.94 μg/g*h in the kidney, and 1.1 μg/g*h in the brain. Similar to this, DEDM’s AUC_0-t_ in the lung was 12.98 μg/g*h, whereas it was 0.87, 0.62, 0.13, 1.34, and 0.87 μg/g*h in the heart, liver, spleen, kidney, and brain, respectively. In comparison to other tissues, the distribution of EDM and DEDM in the lung was significantly higher. This behavior may be related to the 2-hydroxypropyl-cyclodextrin (HPβCD), which is made up of covalently linked glucopyranose rings and results in lung enrichment for both EDM and DEDM. By generating water-soluble inclusions that enclose all or a portion of the pharmaceuticals in the hydrophobic cavities of cyclodextrins, they can offer a way to enhance the solubility of hydrophobic medications (Brewster and Loftsson, 2007). The HPβCD solution exhibits a compatible range of droplet size with pulmonary deposition, sustain-release characteristic in drug absorption, and non-toxic in short-term exposure, according to a number of publications that discuss its applicability for pulmonary delivery (Evrard et al., 2004; Tewes et al., 2008; Thi et al., 2009; Guan et al., 2020). As a result, HPβCD is used as a carrier to prepare inclusion complex for distribution to the lungs (Guan et al., 2020; Su et al., 2020).

It is well acknowledged that the dose, or more precisely the amount of drug distributed in the specific tissues, impacts the drug’s potency and level of toxicity. EDM was highly hepatotoxic, resulting in the production of AST, LDH, ALT, and ALP, according to long-term toxicity experiments (Li et al., 2019). However, when C57BL/6 N mice were given 80 mg/kg of EDM orally, another investigation revealed no discernible hepatotoxicity (Zhang Y. et al., 2018). EDM has a poor bioavailability (Lin et al., 2012), and because of this, its concentration in the liver in some short-time orally administration is so limited that it does not cause hepatotoxicity. But, after repeated administration of the medicine, its accumulation in the tissue may be excessive, resulting in toxic performance.

Crossing the BBB is the main hurdle that needs to be overcome for medications to enter brain tissue. Transmembrane transport is a significant component determining the tissue distribution of pharmaceuticals, particularly for brain tissues with specific barriers. Data from *in vitro* experiments revealed that the permeability coefficient of DEDM in the Caco-2 model was 15 times greater than that of EDM. Similar to how DEDM’s drug concentration in the brain was 6.44 times higher than EDM’s under the same dosage. The primary element causing variances in the capacity of EDM and DEDM to penetrate tissue is the dihedral angle of the molecule stereo-structure. The steric structure of molecules has recently received a lot of attention from academics. Clinical (Lovering et al., 2009) and toxicological (Luker et al., 2011) investigations have shown that compounds with more stereochemical characteristics perform better in terms of selectivity and biological activity (Clemons et al., 2010). The current study’s use of analogs, in particular EDM and DEDM, demonstrates the importance of the dihedral angle of the molecular stereo-structure as a determinant of the indoloquinazoline alkaloids' tissue distribution. These results will help us understand the structure-activity correlations of indoloquinazoline alkaloids in tissue distribution better. They will also help us understand how to change a drug’s structure to improve its qualities and lay the groundwork for future drug development.

## 5 Conclusion

Due to its more flatted dihedral angle, DEDM has a more planar stereo-structure than EDM, which increases its permeability by making it simpler to penetrate the phospholipid bilayer. As a result, DEDM has a higher chance of piercing the BBB and entering the brain.

## Data Availability

The original contributions presented in the study are included in the article/Supplementary Material, further inquiries can be directed to the corresponding authors.
