# Supplementary material for: Differences in tissue distribution ability of evodiamine and dehydroevodiamine are due to the dihedral angle of the molecule stereo-structure
Source: Front Pharmacol. 2023 Apr 6;14:1109279. doi: 10.3389/fphar.2023.1109279 (PMC10117637; doi:10.3389/fphar.2023.1109279)
Supplement: Supplementary file 1 [file DataSheet1.docx]

Supplementary Materials for

**Differences in tissue distribution ability of evodiamine and dehydroevodiamine are due to the dihedral angle of the molecule stereo-structure**

Jie Luo^1,2^, Wen Wen^1,2^, Jie Chen^1,2^, Xiaobo Zeng^1,2^, Shijun Xu^1,2^*^*^* and Ping Wang^1,2^*^*^*

1. School of Pharmacy, Chengdu University of Traditional Chinese Medicine, Chengdu, 611137, PR China
2. Institute of Meterial Medica Integration and Transformation for Brain Disorders, Chengdu University of Traditional Chinese Medicine, Chengdu, Sichuan 611137, PR China

^*^Corresponding authors: Ping Wang and Shijun Xu

E-mail: Wangping.wp@aliyun.com ; [xushijun@cdutcm.edu.cn](mailto:xushijun@cdutcm.edu.cn)

Jie Luo and Wen Wen are considered as joint first authors.

| **Table S1.Multiple reaction monitoring for evodiamine and dehydroevodiamine** | | | | |
| --- | --- | --- | --- | --- |
| Compounp | Precursorion(m/z) | Product ion(m/z) | Fragmentor | Collision energy |
| Evodiamine | 304.3 | 171 | 90 | 18 |
| Dehydroevodiamine | 301.3 | 286 | 140 | 32 |

| **Table S2.The linear regression equation of six alkaloids** | | |
| --- | --- | --- |
| Compounp | Detection range（ug/mL） | Equation |
| Evodiamine | 0.04-2.5 | Y=218.32x-3.9443(r^2^=0.9991) |
| Rutaecarpine | 0.02-1.3 | Y=30.29x+6.1797(r^2^=0.9951) |
| Dehydro evodiamine | 0.23-7.5 | Y=623.06x-286.47(r^2^=0.996) |
| Evolitrine | 0.18-2.9 | Y=21.839x+15.351(r^2^=0.999) |
| hydroxy-evodiamine | 0.06-4.0 | Y=149.39x+33.225(r^2^=0.9995) |
| 1-hydroxyrutaecarpine | 0.03-0.5 | Y=133.05x+6.9883(r^2^=0.997) |

| **Table S3.The nontoxic concentration of six alkaloids** | | |
| --- | --- | --- |
| Compounp | Detection range（uM） | Nontoxic concentration |
| Evodiamine | 0.4-100 | 12.5 |
| Rutaecarpine | 0.4-100 | 100 |
| Dehydroevodiamine | 0.4-100 | 100 |
| Evolitrine | 1.6-200 | 100 |
| hydroxy-evodiamine | 1.6-200 | 200 |
| 1-hydroxyrutaecarpine | 1.6-200 | 100 |

| **Table S4.The linear regression equation of evodiamine** | | |
| --- | --- | --- |
| Tissue | Detection range（ng/mL） | Equation |
| Heart | 1.8-364 | Y=0.0219x+5.0923(r^2^=0.9975) |
| Liver | 1.8-364 | Y=0.0271x-7.4135(r^2^=0.9914) |
| Spleen | 1.8-364 | Y=0.0426x-5.2201(r^2^=0.996) |
| Lung | 1.8-364 | Y=0.0291x+1.6433(r^2^=0.9995) |
| Kindy | 1.8-364 | Y=0.029x+3.8593(r^2^=0.999) |
| Brain | 1.8-364 | Y=0.0216x+6.9058(r^2^=0.9966) |
| Plasma | 1.8-364 | Y=0.0386x+2.8926(r^2^=0.9995) |

| **Table S5.The linear regression equation of dehydroevodiamine** | | |
| --- | --- | --- |
| Tissue | Detection range（ng/mL） | Equation |
| Heart | 1.8-364 | Y=0.0249x+2.3225(r^2^=0.9997) |
| Liver | 1.8-364 | Y=0.0215x+5.6534(r^2^=0.998) |
| Spleen | 1.8-364 | Y=0.0171x+7.9346(r^2^=0.9978) |
| Lung | 1.8-364 | Y=0.016x+9.1799(r^2^=0.9961) |
| Kindy | 1.8-364 | Y=0.0286x-0.7463(r^2^=0.9987) |
| Brain | 1.8-364 | Y=0.0246x+0.9079(r^2^=0.9993) |
| Plasma | 1.8-364 | Y=0.0263x+0.408(r^2^=0.9993) |

| **Table S6. Atomic charge of evodiamine and dehydroevodiamine** | | | |
| --- | --- | --- | --- |
| Named serial number | Atom | evodiaamine | dehydroevodiamine |
| 1 | O | -0.65279 | -0.64556 |
| 2 | C | 0.06166 | 0.21359 |
| 3 | N | -0.40643 | -0.41592 |
| 4 | C | 0.11893 | -0.3274 |
| 5 | C | -0.03056 | -0.15512 |
| 6 | C | -0.17485 | -0.0195 |
| 7 | C | -0.3214 | 0.11616 |
| 8 | C | -0.03107 | -0.0425 |
| 9 | C | 0.45374 | 0.44777 |
| 10 | C | 0.16868 | 0.15918 |
| 11 | C | 0.17538 | -0.07634 |
| 12 | C | -0.08137 | 0.22659 |
| 13 | N | -0.363 | -0.36046 |
| 14 | C | -0.42879 | -0.38701 |
| 15 | C | -0.20556 | -0.19611 |
| 16 | N | -0.56356 | -0.50534 |
| 17 | C | -0.23381 | -0.22797 |
| 18 | C | -0.25321 | -0.14322 |
| 19 | C | -0.14652 | -0.23314 |
| 20 | C | -0.17827 | -0.175 |
| 21 | C | -0.1628 | -0.15886 |
| 22 | C | -0.16338 | -0.17606 |
| 23 | C | -0.18094 | -0.15887 |

| 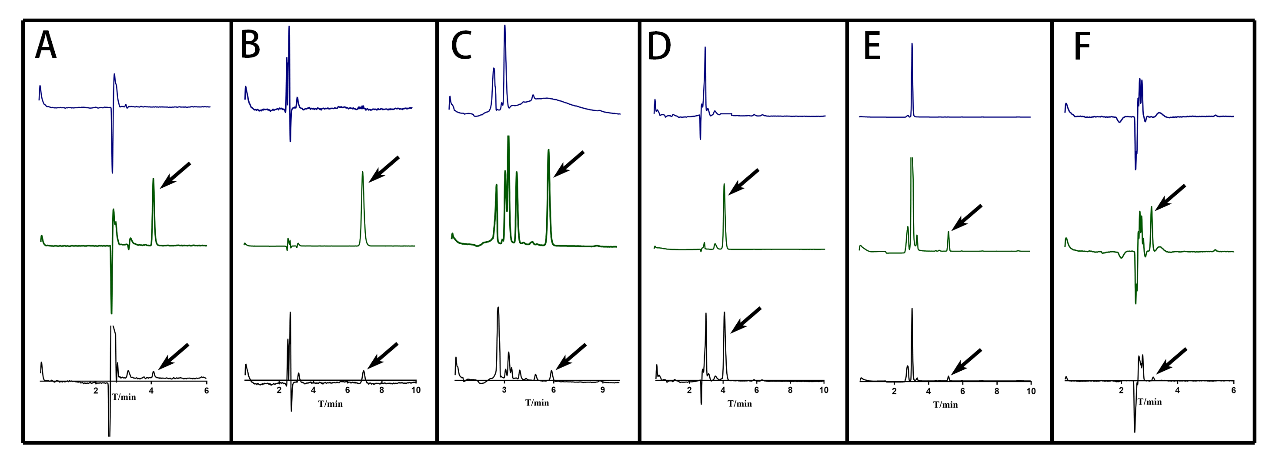 |
| --- |
| **Figure S1** The representative chromatogram of six alkaloids. (**A**) evodiamine (DEM); (**B**) rutaecarpine (RUP); (**C**) dehydroevodiamine (DEDM); (**D**) evolitrine (EVT); (**E**) hydroxyevodiamine (HDEM); (**F**) 1-hydroxyrutaecarpine (1-HRUP). Blue for blank solvent; Green for standard; Black for sample.The retention time were 4.067 minutes for EDM, 6.959 for RUP, 5.869 minutes for DEDM, 4.089 for EVT, 5.210 for HDEM and 3.583 for 1-HRUP, Separately. |

| 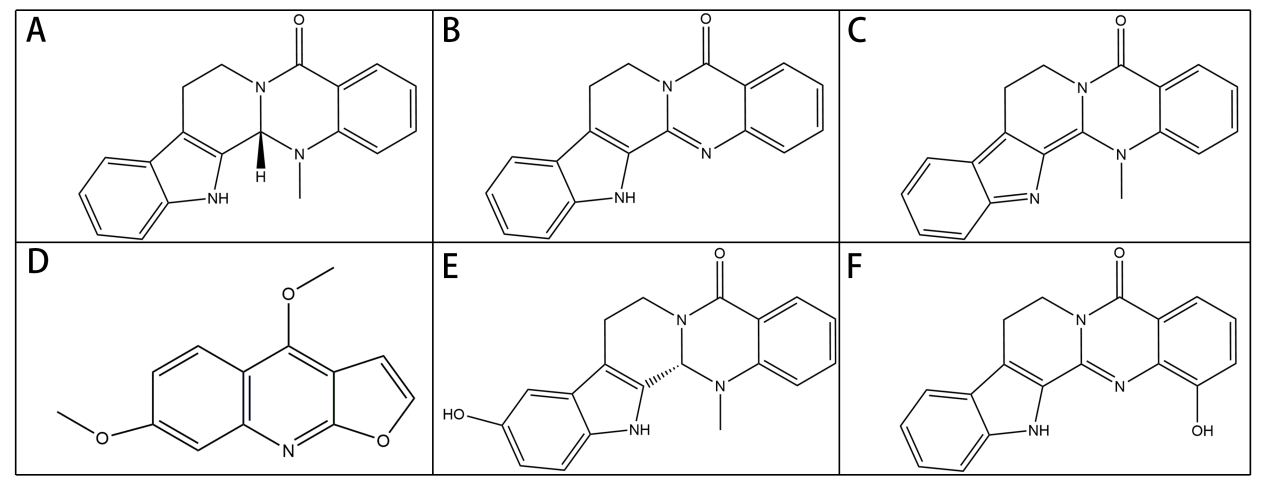 |
| --- |
| **Figure S2** The chemical structures of six alkaloids. (**A**) evodiamine (DEM); (**B**) rutaecarpine (RUP); (**C**) dehydroevodiamine (DEDM); (**D**) evolitrine (EVT); (**E**) hydroxyevodiamine (HDEM); (**F**) 1-hydroxyrutaecarpine (1-HRUP). |

| 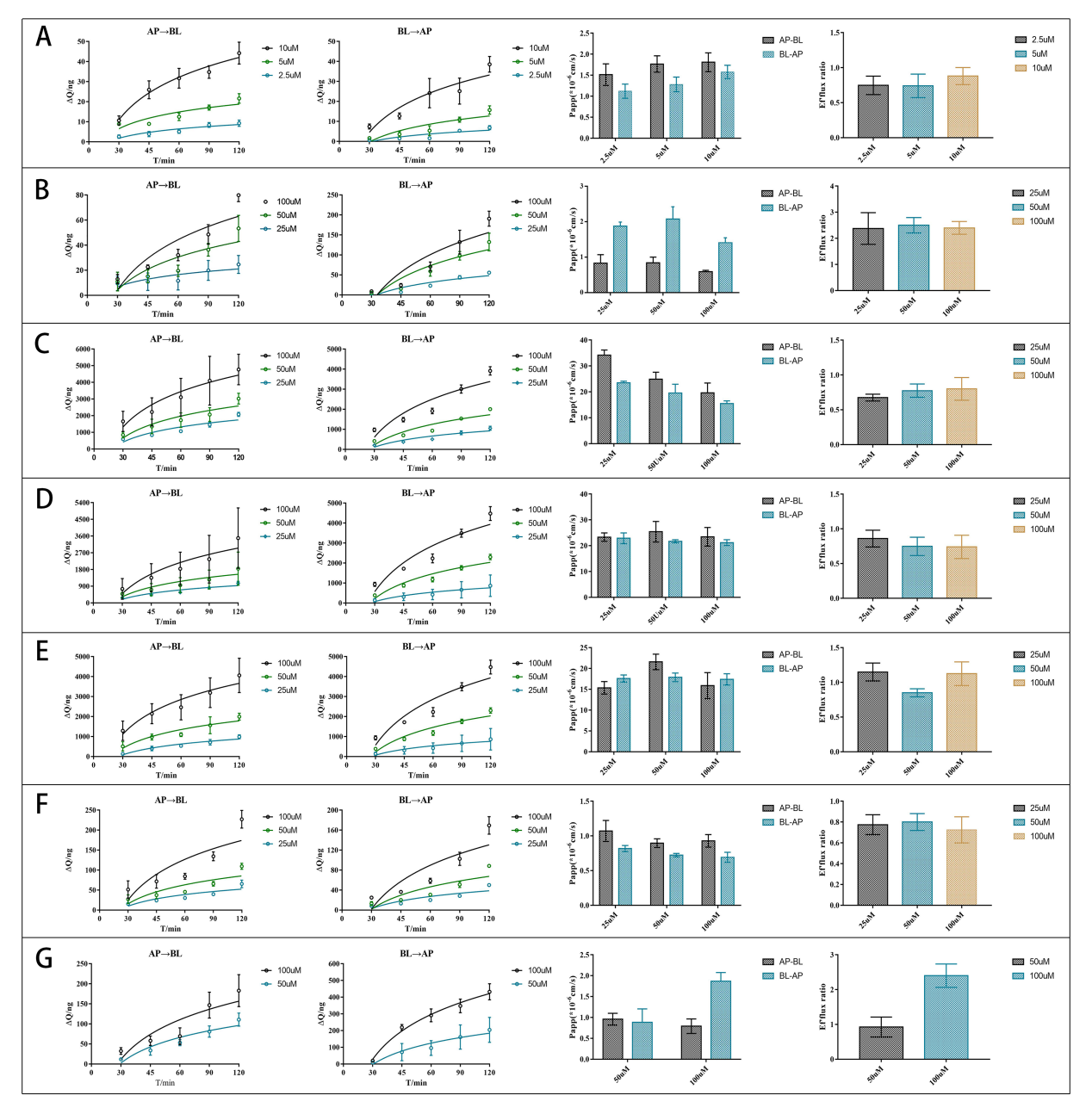 |
| --- |
| **Figure S3** The representative chromatogram of six alkaloids. CaCo-2 cells were cultured in Transwell plate for 21 days to form Caco-2 monolayers. The RUP, DEDM, EVT, HDEM, 1-HRUP (25, 50, and 100 μM) and EDM (2.5, 5, and 10 μM) were added to the donor side, while the receiver chamber contained the corresponding volume of HBSS medium. The sample was collected from each receiver chamber at 0, 30,45, 60, 90, and 120 min. The samples were analysed by high-performance liquid chromatography (HPLC) and calculation apparent permeability coefficients (Papp) and efflux ratio (ER). From the left to right were the accumulation and Papp of drugsacross AP-BL and BL-AP, the efflux ratio of drugs. From the left to right were the accumulation and Papp of marker drugsacross AP-BL and BL-AP, the efflux of drugs; (**A**) evodiamine (DEM); (**B**) rutaecarpine (RUP); (**C**) dehydroevodiamine (DEDM); (**D**) evolitrine (EVT); (**E**) hydroxyevodiamine (HDEM); (**F**) 1-hydroxyrutaecarpine (1-HRUP); (**G**) RUP (25, 100 μM) + Verapamil (100μM); Data are shown as the mean±SD (n=6). |
